# Marine algal flora of Flores and Corvo Islands, Azores

**DOI:** 10.3897/BDJ.9.e60929

**Published:** 2021-02-03

**Authors:** Ana I Azevedo Neto, Manuela I. Parente, Ian Tittley, Robert L. Fletcher, William Farnham, Ana C. Costa, Andrea Z. Botelho, Sandra Monteiro, Roberto Resendes, Pedro Afonso, Afonso C.L. Prestes, Nuno V. Álvaro, David Mila-Figueras, Raul M. A. Neto, José M. N. Azevedo, Ignacio Moreu

**Affiliations:** 1 cE3c - Centre for Ecology, Evolution and Environmental Changes/Azorean Biodiversity Group, Faculdade de Ciências e Tecnologia, Departamento de Biologia, Universidade dos Açores, 9500-321 Ponta Delgada, Açores, Portugal cE3c - Centre for Ecology, Evolution and Environmental Changes/Azorean Biodiversity Group, Faculdade de Ciências e Tecnologia, Departamento de Biologia, Universidade dos Açores 9500-321 Ponta Delgada, Açores Portugal; 2 CIBIO, Centro de Investigação em Biodiversidade e Recursos Genéticos, InBIO Laboratório Associado, Pólo dos Açores, Universidade dos Açores, Faculdade de Ciências e Tecnologia, Departamento de Biologia, 9500-321 Ponta Delgada, Açores, Portugal CIBIO, Centro de Investigação em Biodiversidade e Recursos Genéticos, InBIO Laboratório Associado, Pólo dos Açores, Universidade dos Açores, Faculdade de Ciências e Tecnologia, Departamento de Biologia 9500-321 Ponta Delgada, Açores Portugal; 3 Natural History Museum, Cromwell Road, London, Code SW7 5BD, United Kingdom Natural History Museum Cromwell Road, London, Code SW7 5BD United Kingdom; 4 Institute of Marine Sciences, Department of Biological Sciences, University of Portsmouth, Ferry Road, Eastney, Portsmouth, PO4 9LY, United Kingdom Institute of Marine Sciences, Department of Biological Sciences, University of Portsmouth Ferry Road, Eastney, Portsmouth, PO4 9LY United Kingdom; 5 Faculdade de Ciências e Tecnologia, Departamento de Biologia, Universidade dos Açores, 9500-321 Ponta Delgada, Açores, Portugal Faculdade de Ciências e Tecnologia, Departamento de Biologia, Universidade dos Açores 9500-321 Ponta Delgada, Açores Portugal; 6 IMAR/Okeanos, Departamento de Oceanografia e Pescas, Universidade dos Açores, Rua Prof. Doutor Frederico Machado, 9901-862 Horta, Açores, Portugal IMAR/Okeanos, Departamento de Oceanografia e Pescas, Universidade dos Açores, Rua Prof. Doutor Frederico Machado 9901-862 Horta, Açores Portugal; 7 CCMMG (Centro do Clima Meteorologia e Mudanças Globais) & IITA-A (Instituto de Investigação e Tecnologias Agrárias e do Ambiente), Universidade dos Açores, Faculdade de Ciências Agrárias, Rua Capitão João d’Ávila – Pico da Urze, 9700-042 Angra do Heroísmo, Açores, Portugal CCMMG (Centro do Clima Meteorologia e Mudanças Globais) & IITA-A (Instituto de Investigação e Tecnologias Agrárias e do Ambiente), Universidade dos Açores, Faculdade de Ciências Agrárias, Rua Capitão João d’Ávila – Pico da Urze 9700-042 Angra do Heroísmo, Açores Portugal; 8 NA, NA, Portugal NA NA Portugal

**Keywords:** Macroalgae, Azores, Corvo Island, Flores Island, new records, endemism, native, uncertain, introduced, occurrence data.

## Abstract

**Background:**

The algal flora of the western group of the Azores archipelago (Islands of Flores and Corvo) has attracted the interest of many researchers on numerous past occasions (such as Drouet 1866, Trelease 1897, Gain 1914, Schmidt 1929, Schmidt 1931, Azevedo et al. 1990, Fralick and Hehre 1990, Neto and Azevedo 1990, Neto and Baldwin 1990, Neto 1996, Neto 1997, Neto 1999, Tittley and Neto 1996, Tittley and Neto 2000, Tittley and Neto 2005, Tittley and Neto 2006, Azevedo 1998, Azevedo 1999, Tittley et al. 1998, Dionísio et al. 2008, Neto et al. 2008). Despite this interest, the macroalgal flora of the Islands cannot be described as well-known with the published information reflecting limited collections preformed in short-term visits by scientists. To overcome this, a thorough investigation, encompassing collections and presence data recording, has been undertaken for both the littoral and sublittoral regions, down to a depth of approximately 40 m, covering a relatively large area on both Islands (approximately 143 km^2^ for Flores and 17 km^2^ for Corvo).

This paper lists the resultant taxonomic records and provides information on species ecology and occurrence around both these Islands, thereby improving the knowledge of the Azorean macroalgal flora at both local and regional scales.

**New information:**

For the Island of Flores, a total of 1687 specimens (including some taxa identified only to genus level) belonging to 196 taxa of macroalgae are registered, comprising 120 Rhodophyta, 35 Chlorophyta and 41 Ochrophyta (Phaeophyceae). Of these taxa, 128 were identified to species level (80 Rhodophyta, 22 Chlorophyta and 26 Ochrophyta), encompassing 37 new records for the Island (20 Rhodophyta, 6 Chlorophyta and 11 Ochrophyta); two Macaronesian endemics (*Laurencia viridis* Gil-Rodríguez & Haroun and *Millerella tinerfensis* (Seoane-Camba) S.M.Boo & J.M.Rico); six introduced (the Rhodophyta *Asparagopsis armata* Harvey, *Neoizziella divaricata* (C.K.Tseng) S.-M.Lin, S.-Y.Yang & Huisman and *Symphyocladia marchantioides* (Harvey) Falkenberg; the Chlorophyta Codium fragile subsp. fragile (Suringar) Hariot; and the Ochrophyta *Hydroclathrus tilesii* (Endlicher) Santiañez & M.J.Wynne and *Papenfussiella kuromo* (Yendo) Inagaki); and 14 species of uncertain status (10 Rhodophyta, two Chlorophyta and two Ochrophyta).

For the Island of Corvo, a total of 390 specimens distributed in 56 taxa of macroalgae are registered, comprising 30 Rhodophyta, nine Chlorophyta and 17 Ochrophyta (Phaeophyceae). Whilst a number of taxa were identified only to the genus level, 43 were identified to species level (22 Rhodophyta, eight Chlorophyta and 13 Ochrophyta), comprising 22 new records for the Island (nine Rhodophyta, four Chlorophyta and nine Ochrophyta), two introduced species (the Rhodophyta *Asparagopsis armata* and the Chlorophyta Codium fragile subsp. fragile and seven species of uncertain status (five Rhodophyta and two Ochrophyta).

## Introduction

The Azorean algal flora, considered cosmopolitan, with species shared with Macaronesia, North Africa, the Mediterranean Sea, Atlantic Europe and America (Tittley 2003, Tittley and Neto 2006, Wallenstein et al. 2009), is relatively rich when compared to that of other remote oceanic Islands (Neto et al. 2005, Tittley and Neto 2005, Wallenstein et al. 2009). Around 400 species of marine macroalgae have, to date, been recorded for the isolated mid-Atlantic Azores archipelago (Freitas et al. 2019). These authors, based on extensive analysis encompassing data on brachyurans, polychaetes, gastropods, echinoderms, coastal fishes and macroalgae, suggested that the Azores should be a biogeographical entity of its own and proposed a redefinition of the Lusitanian biogeographical province, in which they recognised four ecoregions: the South European Atlantic Shelf, the Saharan Upwelling, the Azores ecoregion and a new ecoregion herein named Webbnesia, which comprises the archipelagos of Madeira, Selvagens and the Canary Islands. In their paper comparing the Azorean algal flora to that of the new Webbnesia region, they reported that the Canary Islands, with 689 species of marine macroalgae, are by far the most diverse archipelago, followed by the Azores (405), Madeira (396) and Cabo Verde (333). The Selvagens are the least diverse one (295 species). It is worth mentioning that the published information reflects data from only a few of the nine Azorean Islands, since not all of them have been adequately investigated. In the Azores archipelago, São Miguel is by far the Island with the largest amount of research dedicated to the study of its algal flora. The total number of algal species is, at the moment, 260, a number that is likely to increase due to ongoing research by authors of the present paper. Most of the remaining Islands have received less attention. To overcome this and improve the understanding of the archipelago’s macroalgal flora, research has been conducted over the past three decades on all the Islands. Data on the Islands of Pico, Graciosa and Terceira is already available on the recently-published papers (Neto et al. 2020a, Neto et al. 2020b, Neto et al. 2020c). Table 1 summarises the currently-available information.

To provide a better understanding of the archipelago’s seaweed flora, a long term research programme of study has been undertaken, mainly by local investigators into the marine macroalgae flora on several of the less studied Azorean Islands. The present paper presents both physical and occurrence data and information gathered from surveys undertaken on Flores and Corvo Islands mainly by the Island Aquatic Research Group of the Azorean Biodiversity Centre of the University of the Azores (Link: https://ce3c.ciencias.ulisboa.pt/sub-team/island-aquatic-ecology), the BIOISLE, Biodiversity and Islands Research Group of CIBIO-Açores at the University of the Azores (Link: https://cibio.up.pt/research-groups-1/details/bioisle) and the OKEANOS Centre of the University of the Azores (Link: http://www.okeanos.uac.pt). In these surveys, particular attention was given to the small filamentous and thin sheet-like species that are often short-lived and fast-growing and usually very difficult to identify in the wild, without the aid of a microscope and specialised literature in the laboratory.

The present paper aims to provide a valuable marine biological tool for research on systematics, diversity and conservation, biological monitoring, climate change and ecology for academics, students, government, private organisations and the general public.

## General description

### Purpose

In this paper, we present taxonomic records of macroalgae recorded from the Islands of Flores and Corvo and provide general information on their occurrence and distribution. By doing this, we are contributing to address several biodiversity shortfalls (see Cardoso et al. 2011, Hortal et al. 2015), namely, the need to catalogue the Azorean macroalgae (Linnean shortfall) and improve the current information on their local and regional geographic distribution (Wallacean shortfall), as well as on species abundance and dynamics in space (Prestonian shortfall).

## Project description

### Title

Marine algal flora of Flores and Corvo Islands, Azores.

### Personnel

Collections were made and occurrence data recorded over several years (1989 - 2018). Main collectors were Ana Cristina Costa, Ana I Neto, Andrea Z. Botelho, Carolina Arruda, Cláudia Hipólito, Cristiana Figueiredo, David Milla-Figueras, Heather Baldwin, Inês Neto, Joana Michael, José M. N. Azevedo, Ian Tittley, Manuela I. Parente, Marco Henrique, Maria Ana Dionísio, Maria Ventura, Nuno Vaz Álvaro, Patrícia Madeira, Pedro Cerqueira, Raul Neto, Rita Grilo, Rita Norberto, Robert Fletcher, Sandra Monteiro and William Farnham.

Preliminary *in situ* identifications were carried out by: Ana Cristina Costa, Ana I Neto, Andrea Z. Botelho, David Milla-Figueras, Heather Baldwin, Ian Tittley, Manuela I. Parente, Maria Ventura, Rita Grilo, Robert Fletcher and William Farnham.

Ana I. Neto, Andrea Z. Botelho, David Milla-Figueras, Ian Tittley, Manuela I. Parente, Robert Fletcher and William Farnham were responsible for the final species identification.

Voucher specimen management was mainly undertaken by Afonso Prestes, Ana I. Neto, Andrea Z. Botelho, David Milla-Figueras, Eunice Nogueira, Manuela I. Parente, Natália Cabral and Roberto Resendes.

### Study area description

**Figure 1.**
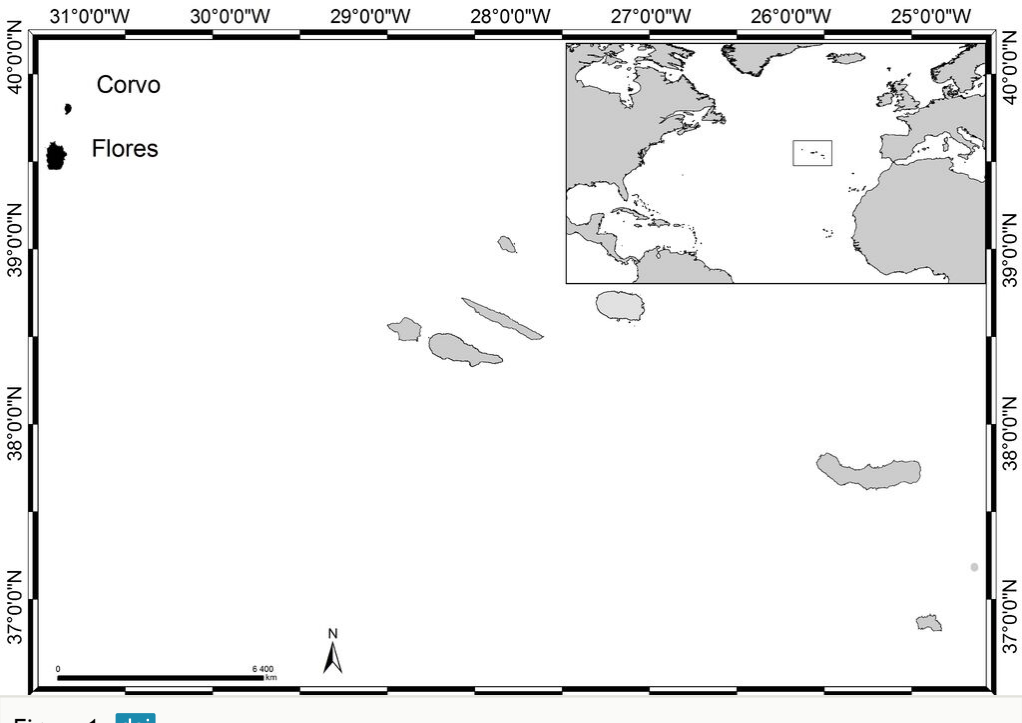
The Azores, its location in the Atlantic and Flores and Corvo Islands highlighted in black (by Nuno V. Álvaro).

The Azores archipelago (38°43′49″N, 27°19′10″W, Fig. 1), comprising nine Islands and several islets, is spread over 500 km, in a WNW direction. The Islands emerged from what is called the Azores Plateau and are located above an active triple junction between three of the world's largest tectonic plates (the North American Plate, the Eurasian Plate and the African Plate, Hildenbrand et al. 2018). Flores and Corvo (in black in Fig. 1), the westernmost Islands of the archipelago, are located in the North American Plate, whereas the remaining Islands are located around the boundary that divides the Eurasian and African Plates (Hildenbrand et al. 2018).

**Figure 2.**
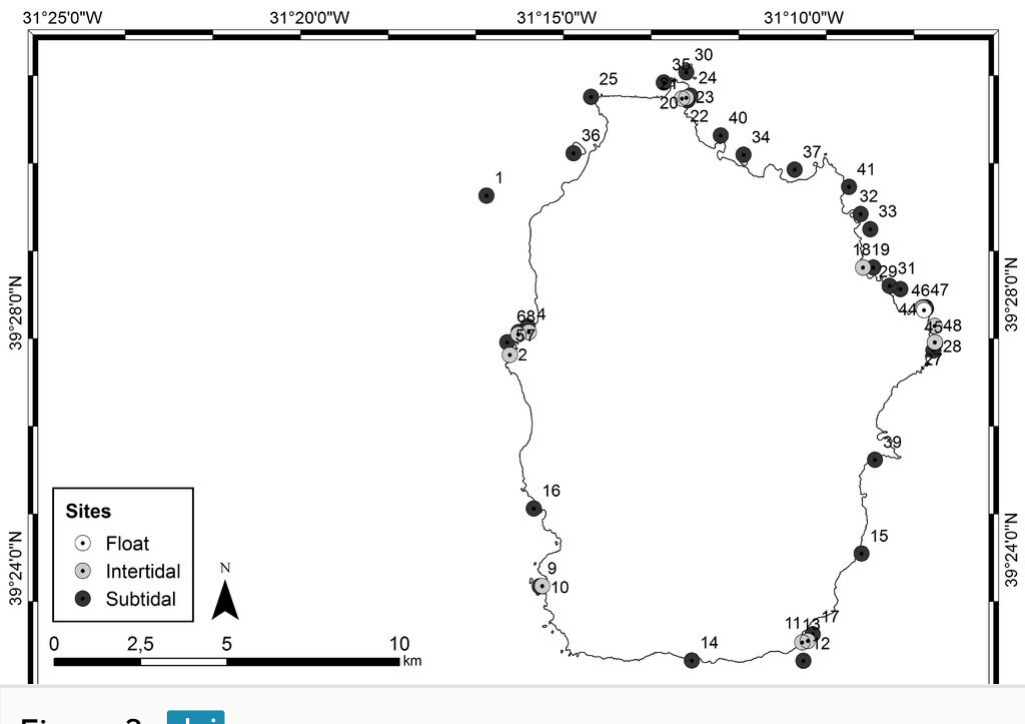
Flores Island showing the sampling locations (by Nuno V. Álvaro).

**Figure 3.**
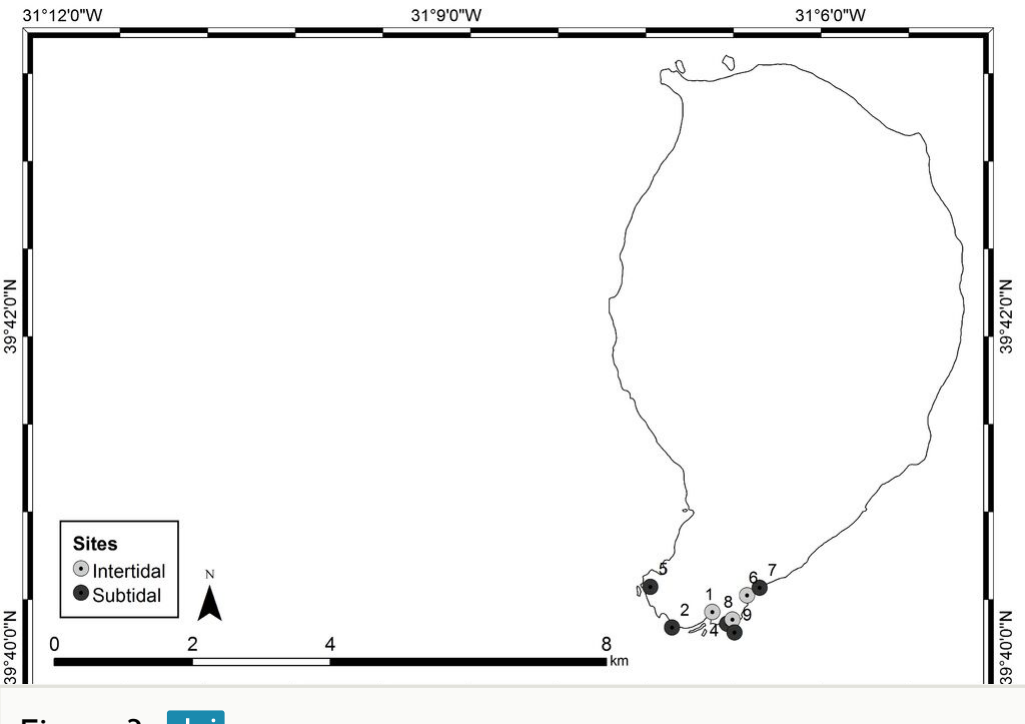
Corvo Island showing the sampling locations (by Nuno V. Álvaro).

The Islands of Flores and Corvo are sub-aerial domains of a large volcanic formation, mostly submarine, implanted on an oceanic crust and aged between 9.0 and 10.0 million years (Ma). Each of these Islands has unique geomorphological characteristics: Flores (39°31'27″N, -31°15'31"W, Fig. 2), of approximately 141 km^2^, is composed of two units, the central massif located in the central plain and the coastal periphery; Corvo, its neighbour (39°43'37"N, -31°7'44"W, Fig. 3), of approximately 17 km^2^, is a crater of a major Plinian eruption and the smallest Island of the Azores archipelago (Azevedo 1999). The climate, as in the remaining Islands, is characterised by regular and abundant rainfall, high levels of relative humidity and persistent winds, mainly during the winter and autumn seasons (Morton et al. 1998). Fog is common and almost permanent at the higher elevations.

Marine action is responsible for the predominance of erosive morphologies in the coastal areas of both Islands, examples of which on Flores (Neto et al. 2008) are: the valleys associated with fluvial erosion (Vales das Lajes and da Fazenda); the coastal or back cliffs (Fajãzinha - Ponta da Fajã); the large marine abrasion platforms (Fajãzinha - Fajã Grande); and the coastal platforms associated with landslides and collapses (Ponta da Fajã).

Owing to the lack of a continental shelf that characterises most volcanic Islands, coastal extension is restricted and deep waters occur within a few kilometres offshore. The tidal range is small (< 2 m, Hidrogrográfico 1981) and coasts are subjected to swell and surge for most of the year.

**Figure 4.**
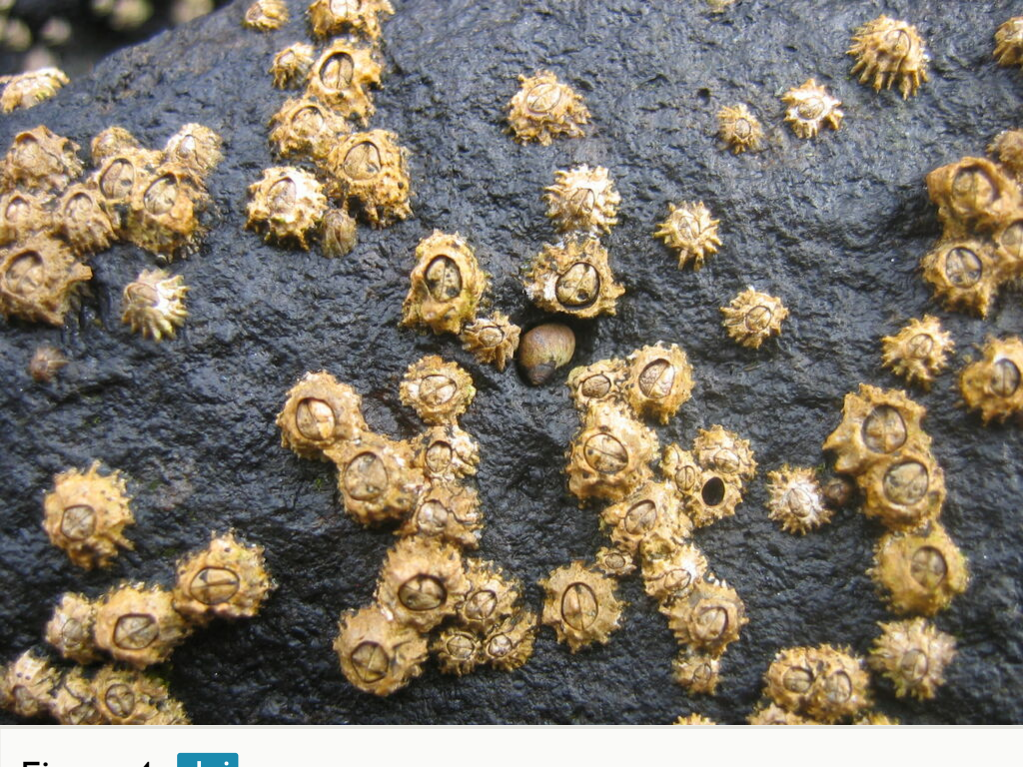
Chthamalid barnacles and littorinids, characteristic species of the Azorean high intertidal level (by the Island Aquatic Ecology Subgroup of cE3c-ABG).

**Figure 5.**
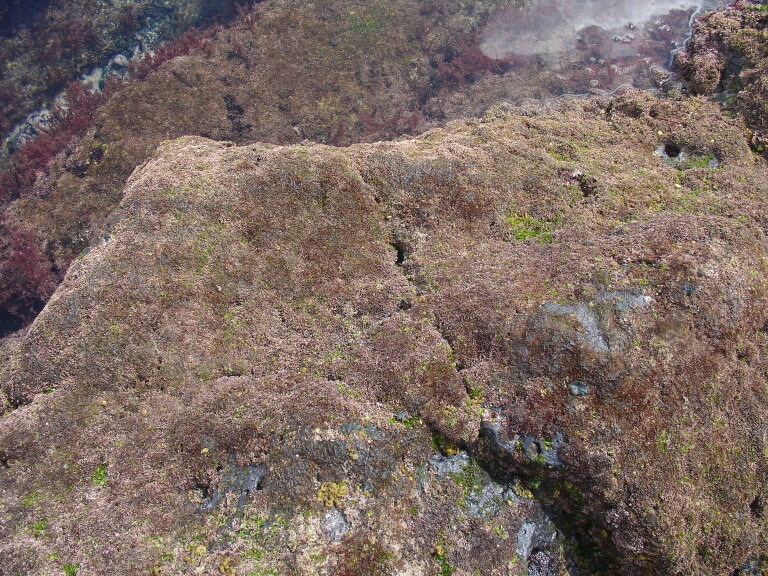
Algal turfs at the low-shore intertidal level (by the Island Aquatic Ecology Subgroup of cE3c-ABG).

**Figure 6.**
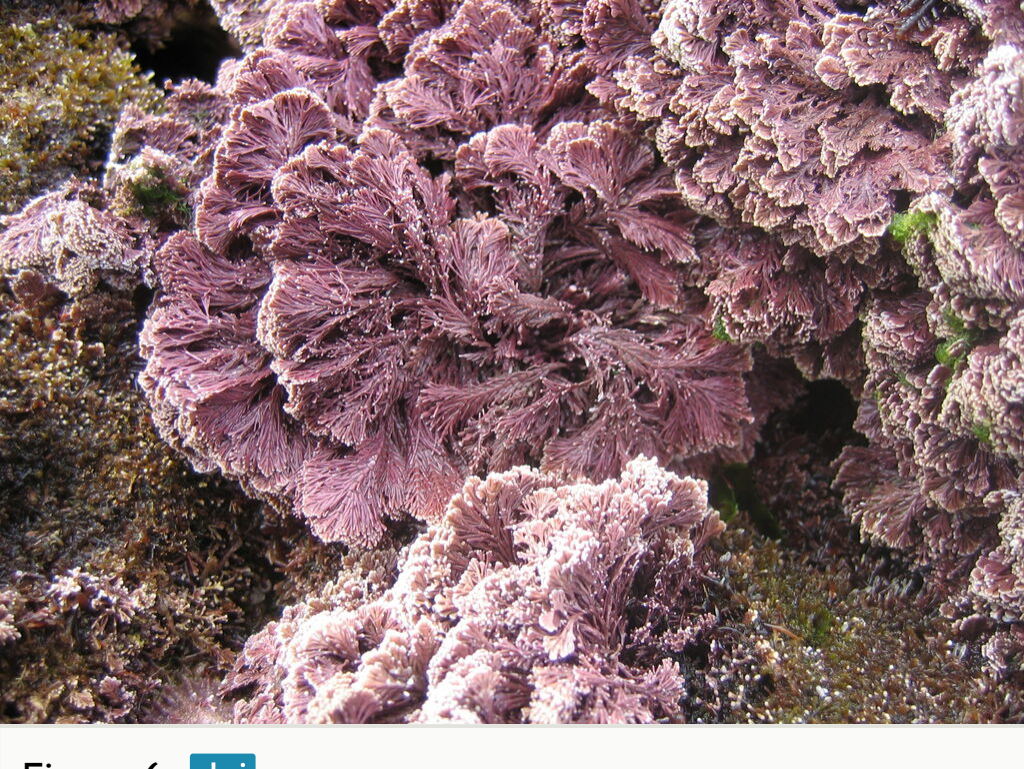
The calcareous frondose alga *Ellisolandia elongata* at the low intertidal level (by the Island Aquatic Ecology Subgroup of cE3c-ABG).

**Figure 7.**
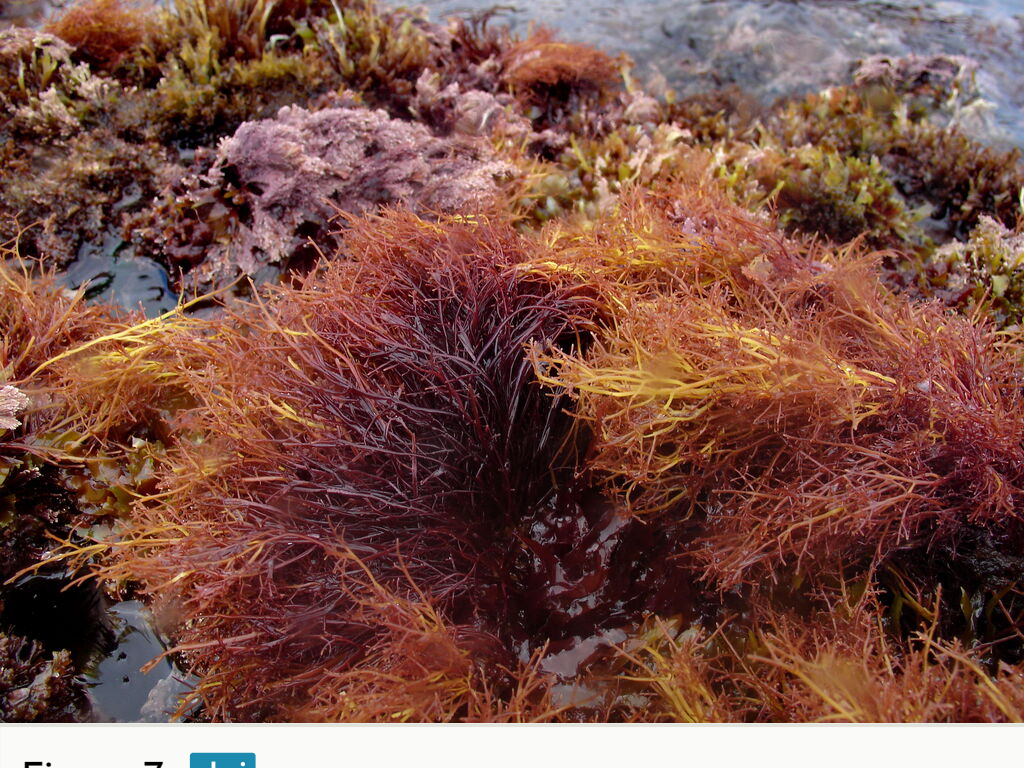
The red agarophyte *Pterocladiella capillacea*, a common species at the low intertidal level (by the Island Aquatic Ecology Subgroup of cE3c-ABG).

**Figure 8.**
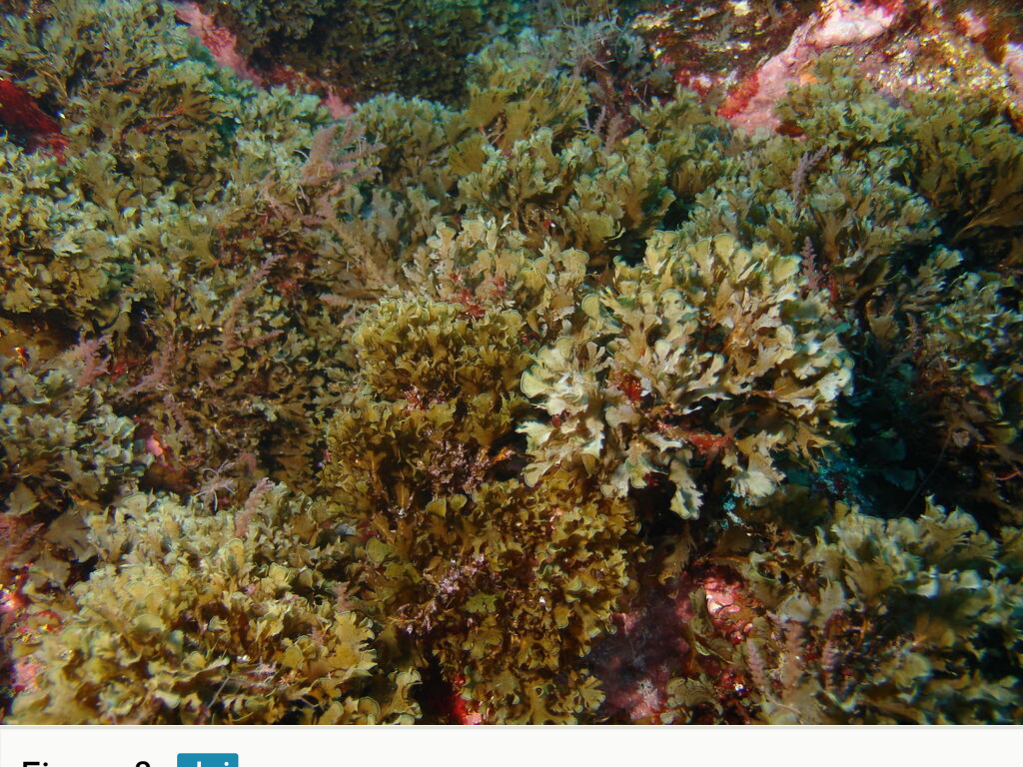
The frondose brown macrophyte *Zonaria tournefortii* at the subtidal level (by the Island Aquatic Ecology Subgroup of cE3c-ABG).

The Islands’ coastline, approximately 72.209 km long on Flores and 19.045 km long on Corvo, is predominantly rocky, subject to strong maritime erosion and presents an irregular slope with extensive and high cliffs cut by waterfalls and streams, alternating with a complex system of bays, rocky beaches and natural terraces (Azevedo 1999). The bottom is mostly made up of irregular rocky bedrock, containing, in some places, pockets of sediment of coarse sand and gravel, alternating with places covered by blocks that rest on either the rocky bed or the sediment. Submerged or semi-submerged caves, arches and tunnels of small amplitude and reduced length are common. As depth increases, the slope decreases, although the bottom is still rocky and uneven. This feature is interrupted by valleys and other structures of smooth to rough relief. The sediment floor in the deepest areas is stable, generally composed of medium and/or coarse sand. From this floor arise small islets with normally vertical walls and low irregular crowns, marked by ridges and valleys (Neto et al. 2008). Along the coastline and islets, natural sheltered habitats (arches and semi-submerged caves, tide pools) create favourable conditions for the growth of juveniles and adults of coastal fish. The constant recycling of nutrients caused by the wave-exposed coasts of these Islands, provides suitable conditions for the occurrence of considerable diversity and abundance of macroinvertebrates and pelagic and benthic fish (Neto et al. 2008). At the foot of the cliffs, the rocky intertidal zone is, as elsewhere in the Azores, dominated by algal communities that form mosaic and/or horizontal bands relative to tide level and are made up of multispecific algal turfs (growth forms of either diminutive algae or diminutive forms of larger species) that carpet the rocks. In the intertidal, a distinct zonation pattern is evident. The higher zone, dominated by invertebrates (littorinids and chthamalid barnacles, Fig. 4), gives rise below to a mid-shore zone covered by algal turfs that create a dense, compact mat 20-30 mm in thickness, Fig. 5). The turf can be monospecific (of either *Caulacanthus ustulatus* (Turner) Kützing, *Centroceras clavulatum* (C. Agardh) Montagne or *Gymnogongrus*) or multispecific and composed by soft algae (e.g. *Centroceras clavulatum*, *Ceramium* and *Chondracanthus*) usually growing as epiphytes over articulate calcareous forms (e.g. *Ellisolandia* and *Jania*). The low-shore zone is mainly dominated by calcareous crusts (first/basal strata), covered by corticated macrophytes, for example, *Ellisolandia elongata* (J.Ellis & Solander) K.R.Hind & G.W.Saunders (Fig. 6) and *Pterocladiella capillacea* (S.G.Gmelin) Santelices & Hommersand (Fig. 7) and, in more exposed locations, *Tenarea tortuosa* (Esper) Me Lemoine (Neto et al. 2008). Seasonally and mainly in spring and summer, the introduced red alga *Asparagopsis armata* occurs often abundantly at this lower intertidal level. Important features and habitats at this shore level are rock pools, occurring in different shapes and sizes and often recreating a shallow subtidal habitat, which contains a rich diversity of marine life. A few shores consist of irregularly rounded boulders or cobbles between which coarse sand or gravel may be retained. Sandy shores are rare (Neto, pers. observ.). The rocky bottoms in the submerged zone are covered by more frondose macrophytes, such as *Pterocladiella capillacea*, *Halopteris filicina* (Grateloup) Kützing, *Dictyota* spp. or *Zonaria tournefortii* (J.V.Lamouroux) Montagne (Fig. 8). At this level, the edible barnacles *Megabalanus azoricus* (Pilsbry, 1916) and/or the limpets *Patella aspera* Röding, 1798 are concentrated in the first few metres, while the slipper lobsters *Scyllarides latus* (Latreille, 1803) or the spiny lobsters *Palinurus elephas* (Fabricius, 1787) are found at greater depths. Several species of fish, such as the blue wrasse *Symphodus caeruleus* (Azevedo, 1999) or the ornate wrasse *Thalassoma pavo* (Linnaeus, 1758), are particularly frequent in shallow rocky areas, whereas other fish take shelter in crevices during the day, such as the morays, *Muraena helena* Linnaeus, 1758 or the forkbeards *Phycis phycis* (Linnaeus, 1766). Still other species roam amongst rocky reefs, such as the parrotfish *Sparisoma cretense* (Linnaeus, 1758), the salemas *Sarpa salpa* (Linnaeus, 1758) and the white sea bream *Diplodus sargus* (Linnaeus, 1758). In the numerous sea caves around Flores and Corvo, the dusky grouper *Epinephelus marginatus* (Lowe, 1834) occurs with an unknown frequency in most of the other Islands (Neto et al. 2008).

In 2007, both Flores and Corvo Islands were recognised by UNESCO as a Biosphere Reserve and thus integrated into the programme “The Man and the Biosphere”. The programme focuses on the ecological, social and economic dimensions of biodiversity loss and uses the World Network of Biosphere Reserves as a vehicle for knowledge sharing, research and monitoring, education and training and participatory decision-making with local communities. The proposed area for the Biosphere Reserve includes the entire emerged land area of the Islands and a surrounding marine zone, covering a total area of 58,619 hectares in Flores and 25,853 hectares in Corvo and incorporating an important diversity of habitats of regional, national and international importance, which includes, for example, areas integrated in the Natura 2000 Network. The inclusion of a vast marine area promotes explicitly, along with conservation, an integrated management practice between terrestrial, coastal and marine environments (Neto et al. 2008).

### Design description

The algae referred to in this paper were collected during field surveys from both the littoral and sublittoral regions down to approximately 40 m on the Islands of Flores and Corvo. Each sampling location was visited several times. On each occasion, a careful and extensive survey was undertaken to provide good coverage of the area. Both presence recording and physical collections were made by walking over the shores or by SCUBA diving. The specimens collected were taken to the laboratory for identification and preservation and the resulting vouchers were deposited in the AZB Herbarium Ruy Telles Palhinha and the Molecular Systematics Laboratory at the Faculty of Sciences and Technology of the University of the Azores.

### Funding

This study was mainly financed by the following projects/scientific expeditions:

- Projects:
  - IASTFC- “Impact Assessment Study for the construction of the Transport Infrastructures of the Islands of Flores and Corvo, Azores - natural environment”, funded by the Azores Regional Government - Regional Secretariat for Tourism and Environment / Regional Environment Directorate, 1990;
  - LFFC- “Littoral flora of the islands of Flores and Corvo: Inventory, ecology and biogeographic affinities”, Government of the Azores - Regional Secretariat for Tourism and Environment / Regional Environment Directorate (GRA-SRTA / DRA), 1995-1999;
  - Project Flores- Biosphere - “Application of Flores Island to a Biosphere Reserve”. Government of the Azores - Regional Secretariat for the Environment and the Sea (GRA-SRAM). 2007-2008;
  - Project MOST - “Application of a model of sustainable tourism to areas of Natura 2000 network in the Azores” (PTDC / AAC-AMB / 104714/2008). Foundation for Science and Technology and the Government of the Azores - Regional Secretariat for the Sea, Science and Technology, Regional Directorate for Sea Affairs (GRA / SRMCT-DRAM), 2010 - 2013;
  - Project PIMA – “Elaboration of the implementation program of the Marine Strategy Framework Directive - Marine Invasion Program in the Azores” (3 /DRAM /2015). Government of the Azores - Regional Secretariat for the Sea, Science and Technology, Regional Directorate for Sea Affairs (GRA / SRMCT-DRAM), 2015;
  - Project BALA – “Elaboration of the implementation program of the marine strategy framework directive - biodiversity of the coastal environments of the Azores” (2 /DRAM /2015). Government of the Azores - Regional Secretariat for the Sea, Science and Technology, Regional Directorate for Sea Affairs (GRA / SRMCT-DRAM), 2015;
  - Project “ACORES-01-0145-FEDER-000072 - AZORES BIOPORTAL – PORBIOTA. Operational Programme Azores 2020 (85% ERDF and 15% regional funds);
- Scientific Expeditions and campaigns:
  - “FLORES/89”, organised by the Biology Department of the University of the Azores, Flores Island, Azores, July 1989;
  - “Earthwatch FLORES/95”, a joint organisation of the Marine Biology Section of the Biology Department of the University of the Azores and the Natural History Museum (London), co-funded by the Earthwatch International and developed under the project LFFC, July – August 1995;
  - “FLORES & CORVO/99”, developed under the project LFFC, July 1999;
  - “FLORES & CORVO/2007”, XIII Scientific Expedition of the Biology Department of the University of the Azores, Islands of Flores and Corvo, July 2007;
  - “MOST”, under the project MOST, 2011-2013;
  - “PIMA/BALA”, under the projects PIMA and BALA, 2015;
- Other funds:
  - Portuguese National Funds, through FCT– Fundação para a Ciência e a Tecnologia, within the projects UID/BIA/00329/2013, 2015-2019, UID/BIA/00329/2020-2023 and UID/BIA/50027/2019 and POCI-01-0145-FEDER-006821;
  - ERDF funds through the Operational Programme for Competitiveness Factors – COMPETE;
  - Portuguese Regional Funds, through DRCT - Regional Directorate for Science and Technology, within several projects, 2019 and 2020 and SRMCT / DRAM - Regional Secretariat for the Sea, Science and Technology, Regional Directorate for Sea Affairs;
  - CIRN/DB/UAc (Research Centre for Natural Resources, Universidade dos Açores, Departamento de Biologia);
  - CIIMAR (Interdisciplinary Centre of Marine and Environmental Research, Porto, Portugal).

## Sampling methods

### Study extent

This study covers a relatively large area, of approximately 143 km^2^ on Flores and 17 km^2^ on Corvo, covering littoral and sublittoral levels down to approximately 40 m around the Islands (Tables 2, 3, Figs 2, 3).

### Sampling description

**Figure 9.**
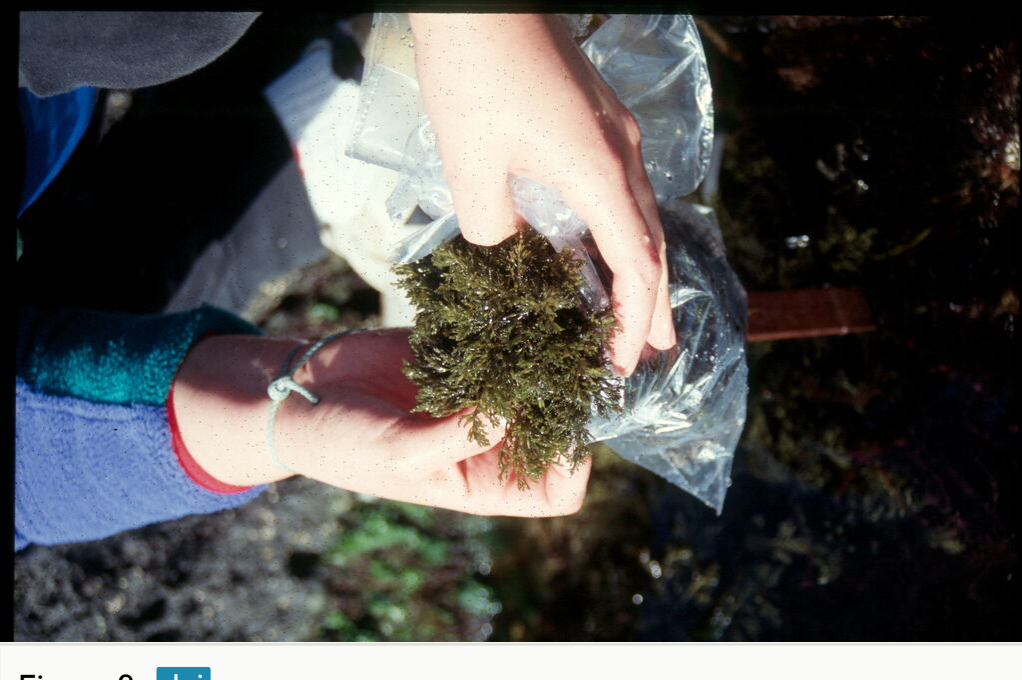
Collecting macroalgae at the rocky intertidal (by the Island Aquatic Ecology Subgroup of cE3c-ABG).

**Figure 10.**
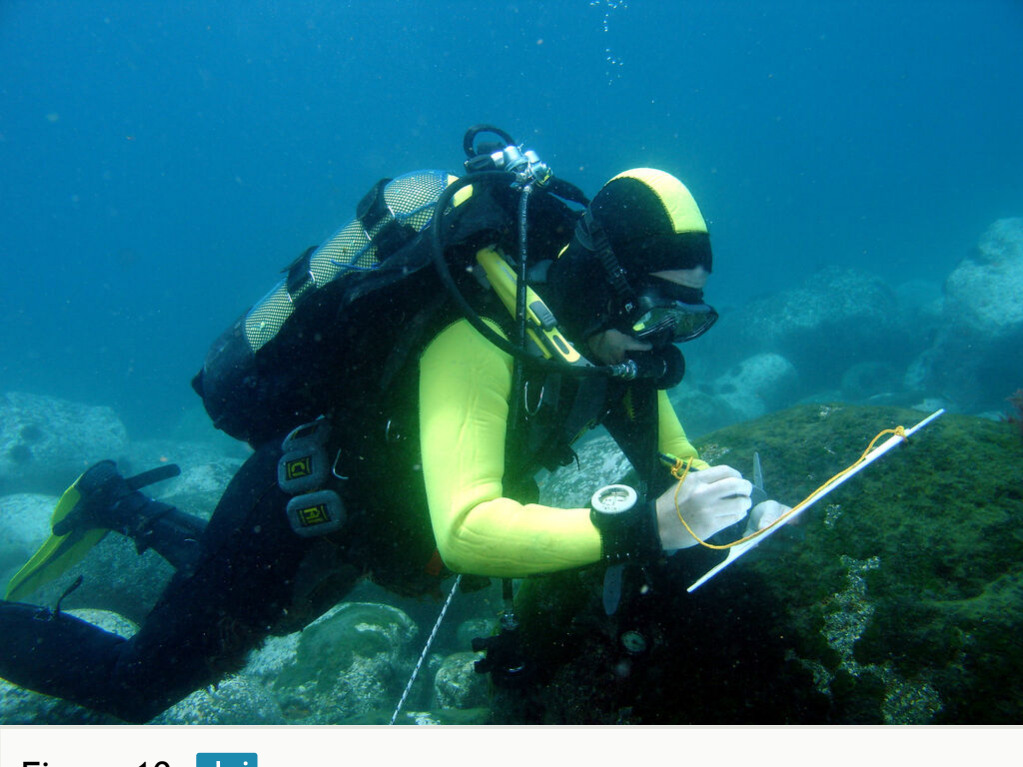
Quantitative recording of the presence and coverage of macroalgal species at the subtidal rocky habitat (by the Island Aquatic Ecology Subgroup of cE3c-ABG).

Intertidal collections were made during low tide by walking over the shores. Subtidal collections were made by SCUBA diving around the area. Sampling involved specimen collecting and species presence recording. For the former, at each location, samples were obtained by scraping from the surface one or two specimens of all the observed species and then placing them into labelled bags (Fig. 9). Species recording data was gathered by registering all species present in the sampled locations visited (Fig. 10).

### Quality control

Each sampled taxon was identified by trained taxonomists and involved morphological and anatomical observations of whole specimens by eye and/or of histological preparations under microscopes to determine the main diagnostic features of each species, as described in literature.

### Step description

Specimens were sorted and studied in the laboratory, following standard procedures used in macroalgae identification.

Species identification was usually based on a combination of morphological, anatomical and reproductive features. For small and simple thalli, this required observing the entire thallus with the unaided eye and/or using dissecting and compound microscopes. For larger and more complex algae, investigation of the thallus anatomy required histological procedures (longitudinal and transverse sections) or squashed preparations of mucilaginous thalli, sometimes after staining, to observe vegetative and reproductive structures and other diagnostic features.

The mixed nature of the Azorean algal flora with components from several geographical regions cause difficulties in species identification. Floras and keys for the North Atlantic, Tropical Atlantic and Western Mediterranean were used (e.g. Schmidt 1931, Taylor 1967, Taylor 1978, Levring 1974, Dixon and Irvine 1977, Lawson and John 1982, Irvine 1983, Gayral and Cosson 1986, Fletcher 1987, Afonso-Carrillo and Sansón 1989, Burrows 1991, Boudouresque et al. 1992, Cabioc'h et al. 1992, Maggs and Hommersand 1993, Irvine and Chamberlain 1994, Brodie et al. 2007, Lloréns et al. 2012, Rodríguez-Prieto et al. 2013).

For more critical and taxonomically-difficult taxa, specimens were taken to the Natural History Museum (London) for comparison with collections there.

A reference collection was made for all collected specimens by assigning them a herbarium code number and depositing them at the AZB Herbarium Ruy Telles Palhinha and the Molecular Systematics Laboratory, University of Azores. Depending on the species and on planned further research, different types of collections were made, namely (i) liquid collections using 5% buffered formaldehyde seawater and then replacing it by the fixing agent Kew (Bridsen and Forman 1999); (ii) dried collections, either by pressing the algae (most species) as described by Gayral and Cosson (1986) or by letting them air dry (calcareous species); and (iii) silica gel collections for molecular studies.

Nomenclatural and taxonomic status used here follow *Algaebase* (Guiry and Guiry 2020). The database was organised on FileMaker Pro.

## Geographic coverage

### Description

**Flores Island Description**: Azores, Portugal (approximately 39°31'27″N, -31°15'31"W);

**Coordinates**: 39.524201 and 39.37521 Latitude; -31.258622 and -31.124496 Longitude.

**Corvo Island Description**: Azores, Portugal (approximately 39°43'37"N, -31°7'44"W).

**Coordinates**: 39.726829 and 39.669576 Latitude; -31.12899 and -31.082546 Longitude.

## Taxonomic coverage

### Description

All macroalgae were identified to genus or species level. For Flores, a total of 196 taxa were identified belonging to 24 orders and 54 families, distributed in the phyla Rhodophyta (14 orders and 33 families), Chlorophyta (three orders and nine families) and Ochrophyta (seven orders and 12 families). For Corvo, a total of 56 taxa were identified belonging to 16 orders and 29 families, distributed in the phyla Rhodophyta (seven orders and 16 families), Chlorophyta (three orders and four families) and Ochrophyta (six orders and nine families).

## Temporal coverage

### Notes

The sampling was performed on several occasions between 1989 and 2018.

## Collection data

### Collection name

AZB | Marine macroalgae collection of Flores and Corvo Islands (Azores)-Expedition Flores/89; AZB | Marine macroalgae collection of Flores and Corvo Islands (Azores)-Expedition Earthwatch Flores/95; AZB | Marine macroalgae collection of Flores and Corvo Islands (Azores)-Expedition Flores & Corvo/99; Marine macroalgae collection of Flores and Corvo Islands (Azores)-Expedition Flores & Corvo/2007; AZB | Marine macroalgae collection of Flores and Corvo Islands (Azores)-Occasional sampling; AZB | Marine macroalgae collection of Flores and Corvo Islands (Azores)-Occasional sampling; Marine macroalgae occurrence of Flores and Corvo Islands (Azores)-Expedition Flores & Corvo/99; Marine macroalgae occurrence of Flores and Corvo Islands (Azores)-Project MOST; Marine macroalgae occurrence of Flores and Corvo Islands (Azores)-Campaign PIMA/BALA; Marine macroalgae occurrence of Flores and Corvo Islands (Azores)-Occasional sampling.

### Collection identifier

33967202-6b10-4182-99d2-621d594572cc; cd4c8dd8-49f7-4318-9b3d-c78aaec53c2d; 93772fb0-339a-4081-b742-a101ca66c019; a7ca4500-9608-44eb-9269-528a40264071; 1a7a0a41-5a5c-460c-815d-0c3503a5a2ea; cfc9d276-6d4e-4cc3-8f40-be9c3e5ba6e9; 434097ea-bac3-49ac-9f5a-3aa9b6c10503; db4e55cc-1401-4b1c-9343-fc2a3e27e473; 29ca7edc-3911-4c59-9722-c9aba69ca506; 153bd328-1e16-4e9e-8dc8-56994c25fb31.

### Parent collection identifier

AZB Herbarium Ruy Telles Palhinha, Faculty of Sciences and Technology of the University of the Azores; AZB Herbarium Ruy Telles Palhinha, Faculty of Sciences and Technology of the University of the Azores; AZB Herbarium Ruy Telles Palhinha, Faculty of Sciences and Technology of the University of the Azores; Expedition Flores & Corvo/2007 Macroalgae collection, Faculty of Sciences and Technology of the University of the Azores; AZB Herbarium Ruy Telles Palhinha, Faculty of Sciences and Technology of the University of the Azores; AZB Herbarium Ruy Telles Palhinha, Faculty of Sciences and Technology of the University of the Azores; Not applicable; Not applicable; Not applicable; Not applicable.

### Specimen preservation method

All specimens were preserved as follows: air dry, dried and pressed; liquid (formalin; fixing agent Kew), silica.

### Curatorial unit

AZB Herbarium Ruy Telles Palhinha, Faculty of Sciences and Technology of the University of the Azores.

## Usage licence

### Usage licence

Creative Commons Public Domain Waiver (CC-Zero)

## Data resources

### Data package title

Marine algal flora of Flores and Corvo Islands, Azores

### Resource link

http://ipt.gbif.pt/ipt/resource?r=flores-corvo_seaweed_flora

### Alternative identifiers

http://ipt.gbif.pt/ipt/resource?r=flores-corvo_seaweed_flora

### Number of data sets

1

### Data set 1.

#### Data set name

Marine algal flora of Flores and Corvo Islands, Azores

#### Data format

Darwin Core Archive

#### Number of columns

49

#### Character set

UTF-8

#### Download URL

http://ipt.gbif.pt/ipt/archive.do?r=flores-corvo_seaweed_flora

#### Data format version

1.3

#### Description

This data paper presents physical and occurrence data from macroalgal surveys undertaken on Flores and Corvo Islands between 1989 and 2018 (Neto et al. 2020d). The dataset submitted to GBIF is structured as a sample event dataset, with two tables: event (as core) and occurrences. The data in this sampling event resource have been published as a Darwin Core Archive (DwCA), which is a standardised format for sharing biodiversity data as a set of one or more data tables. The core data table contains 90 records (eventID). The extension data table has 2077 occurrences. An extension record supplies extra information about a core record. The number of records in each extension data table is illustrated in the IPT link. This IPT archives the data and thus serves as the data repository. The data and resource metadata are available for downloading in the downloads section.

**Data set 1.** 

Column label	Column description
eventID	Identifier of the event, unique for the dataset
country	Country of the sampling site
countryCode	Code of the country where the event occurred
stateProvince	Name of the region
island	Name of the island
municipality	Name of the municipality
locality	Name of the locality
locationID	Identifier of the location
decimalLatitude	The geographic latitude of the sampling site
decimalLongitude	The geographic longitud of the sampling site
geodeticDatum	The spatial reference system upon which the geographic coordinates are based
coordinateUncertaintyInMetres	The horizontal distance (in metres) from the given decimalLatitude and decimalLongitude describing the smallest circle containing the whole of the Location
eventDate	Time interval when the event occurred
year	The year of the event
samplingProtocol	Sampling method used during an event
locationRemarks	Zonation level
minimumDepthInMetres	The minimum depth in metres where the specimen was found
maximumDepthInMetres	The maximum depth in metres where the specimen was found
eventRemarks	Notes about the event
occurrenceID	Identifier of the record, coded as a global unique identifier
institutionID	The identifier for the institution having custody of the object or information referred to in the record
institutionCode	The acronym of the institution having custody of the object or information referred to in the record
collectionID	An identifier of the collection to which the record belongs
collectionCode	The name of the collection from which the record was derived
datasetName	The name identifying the dataset from which the record was derived
eventID	Identifier of the event, unique for the dataset
kingdom	Kingdom name
phylum	Phylum name
class	Class name
order	Order name
family	Family name
genus	Genus name
specificEpithet	The name of the first or species epithet of the scientificName
infraspecificEpithet	The name of the lowest or terminal infraspecific epithet of the scientificName, excluding any rank designation
acceptedNameUsage	The specimen accepted name, with authorship
previousIdentifications	Previous name of the specimen, with authorship
scientificName	The name without authorship applied on the first identification of the specimen
scientificNameAuthorship	The authorship information for the scientificName formatted according to the conventions of the applicable nomenclaturalCode
taxonRank	The taxonomic rank of the most specific name in the scientificName
basisOfRecord	The specific nature of the data record
habitat	Description of the habitat where the specimen was found
recordedBy	Person(s) responsible for sampling
catalogNumber	Identifying code for a unique sample lot in a biological collection
identifiedBy	Person(s) responsible for taxa identification
type	The nature of the resource
preparations	The preservation method used for the specimen
establishmentMeans	The establishment status of the organism in the study region
occurrenceRemarks	New record status assignment
license	Reference to the licence under which the record is published

## Additional information

This paper accommodates the 1687 specimens of macroalgae recorded from Flores Island in 196 taxa comprising 128 confirmed species and 68 taxa identified only to generic level. The confirmed species (Tables 4, 5) include 80 Rhodophyta, 22 Chlorophyta and 26 Ochrophyta (Phaeophyceae). Of these, 37 species are newly recorded for the Island (20 Rhodophyta, six Chlorophyta and 11 Ochrophyta). Most species are native, including the two Macaronesian endemics (*Laurencia viridis* and *Millerella tinerfensis*. Six species are introductions to the algal flora (the Rhodophyta *Asparagopsis armata*, *Neoizziella divaricata* and *Symphyocladia marchantioides*; the Chlorophyta Codium fragile subsp. fragile; and the Ochrophyta *Hydroclathrus tilesii* and *Papenfussiella kuromo*). Fourteen species are uncertain in status (10 Rhodophyta, two Chlorophyta and two Ochrophyta).

Many species were only sporadically recorded on Flores, but 19 were commonly found around the Island and occurred quite abundantly in some locations, namely: the Rhodophyta *Acrosorium ciliolatum* (Harvey) Kylin, *Asparagopsis armata, A. taxiformis* (Delile) Trevisan, *Platoma cyclocolpum* (Montagne) F.Schmitz, *Plocamium cartilagineum* (Linnaeus) P.S.Dixon, *Pterocladiella capillacea* and *Sphaerococcus coronopifolius* Stackhouse; the Chlorophyta *Anadyomene stellata* (Wulfen) C.Agardh, *Cladophora prolifera* (Roth) Kützing, *Codium adhaerens* C.Agradh, *Microdictyon umbilicatum* (Velley) Zanardiniand *Ulva rigida* C.Agardh; and the Ochrophyta *Cladostephus spongiosus* (Hudson) C.Agardh, *Colpomenia sinuosa* (Mertens ex Roth) Derbès & Solier in Castagne, *Halopteris filicina*, *Halopteris scoparia* (Linnaeus) Sauvageau, *Padina pavonica* (Linnaeus) Thivy, *Zanardinia typus* (Nardo) P.C.Silva and *Zonaria tournefortii*.

For the Island of Corvo, this paper accommodates the 390 specimens of macroalgae recorded in 56 taxa comprising 43 confirmed species and 13 taxa identified only to genus level. The confirmed species (Tables 6, 7) include 22 Rhodophyta, eight Chlorophyta and 13 Ochrophyta (Phaeophyceae). Of these, 22 species are newly recorded to the Island (nine Rhodophyta, four Chlorophyta and nine Ochrophyta). Most species are native, two represent introductions to the algal flora of the Azores (the Rhodophyta *Asparagopsis armata* and the Chlorophyta Codium fragile subsp. fragile) and seven have an uncertain status (five Rhodophyta and two Ochrophyta).

Nine species were commonly found, some abundantly in some locations, namely: the Rhodophyta *Acrosorium ciliolatum, Asparagopsis armata*, *A. taxiformis*; the Chlorophyta *Microdictyon umbilicatum*; and the Ochrophyta *Colpomenia sinuosa*, *Halopteris filicina*, *H. scoparia*, *Padina pavonica* and *Zonaria tournefortii*.

A mismatch regarding the GBIF backbone taxonomy of some of the macroalgae species names was identified as detailed in Suppl. material 1.

## Supplementary Material

543F88A7-30F5-5B3F-90D3-ACF600781804 10.3897/BDJ.9.e60929.suppl1 Supplementary material 1 DP-FLOR+COR-id_15074_normalized.csv Data type Macroalgae taxonomic mismatching Brief description GBIF does not have the more actualised nomenclature for some of the macroalgae species names. Therefore, the matching tools of its platform were applied to the species list, as required by Pensoft's data auditor, to identify the problematic taxonomic situations. The resulting file (DP-FLOR+COR-id_15074_normalized.csv) is included here, since the names will not be immediately updated in the GBIF Taxonomic Backbone. A request was already sent to GBIF helpdesk to solve this situation. File: oo_474413.csv https://binary.pensoft.net/file/474413 Ana I Neto

## Figures and Tables

**Table 1.** Number of macroalgal species on the Azorean Islands (Neto et al. 2020b, Neto et al. 2020c, Neto et al. 2020d and authors' unpublished data).

Phyllum	Santa Maria	São Miguel	Terceira	Graciosa	São Jorge	Pico	Faial	Flores	Corvo
Rhodophyta	68	168	73	126	35	142	59	59	13
Chlorophyta	20	39	24	31	17	41	16	16	2
Ochrophyta	28	53	16	38	10	42	8	16	4
Total	116	260	113	195	62	225	83	91	19

**Table 2.** Information and location of the sampling sites on Flores Island.

Location Nо	Location ID	Municipality	Locality	Latitude / Longitude	Littoral zone
1	FLO_bris		Baixa Rasa do Ilhéu	39,495215; -31,274644	Subtidal
2	FLO_L_FGem	Lajes	Fajã Grande | Entre-marés	39,453485; -31,267758	Intertidal
3	FLO_L_FGprb	Lajes	Fajã Grande | Porto de Recreio | Baía	39,460831; -31,261651	Subtidal
4	FLO_L_FGprem	Lajes	Fajã Grande | Porto de Recreio | Entre-marés	39,459356; -31,261244	Intertidal
5	FLO_L_FGpvb	Lajes	Faja Grande | Porto Velho | Baía	39,456795; -31,268607	Subtidal
6	FLO_L_FGpvb	Lajes	Faja Grande | Porto Velho | Baía	39,458818; -31,264851	Intertidal
7	FLO_L_FGpve	Lajes	Fajã Grande | Porto Velho | Enseada	39,459471; -31,264743	Subtidal
8	FLO_L_FGpvem	Lajes	Faja Grande | Porto Velho | Entre-marés	39,458818; -31,264851	Intertidal
9	FLO_L_Ls	Lajes	Lajedo	39,392978; -31,259311	Subtidal
10	FLO_L_Lnt	Lajes	Lajedo | Nascente termal	39,393021; -31,258356	Intertidal
11	FLO_L_Lapem	Lajes	Lajes | Atrás do Porto | Entre-marés	39,377324; -31,169366	Intertidal
12	FLO_L_Laps	Lajes	Lajes | Atrás do Porto | Subtidal	39,372111; -31,17103	Subtidal
13	FLO_L_Lem	Lajes	Lajes | Entre-marés	39,376978; -31,171336	Intertidal
14	FLO_L_Flvs	Lajes	Lajes | Fajã de Lopo Vaz	39,372801; -31,208518	Subtidal
15	FLO_L_Fpls	Lajes	Lajes | Fazenda | Porto da Lomba	39,399797; -31,150731	Subtidal
16	FLO_L_Ms	Lajes	Lajes | Mosteiro	39,413261; -31,260714	Subtidal
17	FLO_L_Lp	Lajes	Lajes | Porto	39,379015; -31,167686	Subtidal
18	FLO_SC_CAb	Santa Cruz	Cedros | Alagoa | Baía	39,474441; -31,144853	Subtidal
19	FLO_SC_CAem	Santa Cruz	Cedros | Alagoa | Entre-marés	39,474473; -31,148271	Intertidal
20	FLO_SC_PDapem	Santa Cruz	Ponta Delgada | Atrás do Porto | Entre-marés	39,519728; -31,206613	Intertidal
21	FLO_SC_PDaps	Santa Cruz	Ponta Delgada | Atrás do Porto | Subtidal	39,519568; -31,206579	Subtidal
22	FLO_SC_PDpem	Santa Cruz	Ponta Delgada | Porto | Entre-marés	39,519473; -31,208125	Intertidal
23	FLO_SC_PDpes	Santa Cruz	Ponta Delgada | Porto | Este	39,519017; -31,206235	Subtidal
24	FLO_SC_PDpos	Santa Cruz	Ponta Delgada | Porto | Oeste	39,520223; -31,205269	Subtidal
25	FLO_SC_PDFAs	Santa Cruz	Farol de Albernaz	39,520461; -31,238744	Subtidal
26	FLO_SC_SCaps	Santa Cruz	Santa Cruz | Atrás do porto	39,452411; -31,125155	Subtidal
27	FLO_SC_SCapvem	Santa Cruz	Santa Cruz | Atrás do porto velho	39,454593; -31,124608	Intertidal
28	FLO_SC_SCapvem	Santa Cruz	Santa Cruz | Atrás do porto velho | Entre-marés	39,454593; -31,124608	Intertidal
29	FLO_SC_SCbvs	Santa Cruz	Santa Cruz | Baixa vermelha	39,46859; -31,135821	Subtidal
30	FLO_SC_SCbpds	Santa Cruz	Santa Cruz | Baixas de Ponta Delgada	39,526318; -31,206453	Subtidal
31	FLO_SC_SCfs	Santa Cruz	Santa Cruz | Fazenda	39,469496; -31,139423	Subtidal
32	FLO_SC_SCiars	Santa Cruz	Santa Cruz | Ilhéu de Álvaro Rodrigues	39,488436; -31,148651	Subtidal
33	FLO_SC_SCigs	Santa Cruz	Santa Cruz | Ilhéu do Garajau	39,48444; -31,145556	Subtidal
34	FLO_SC_SCias	Santa Cruz	Santa Cruz | Ilhéu dos Abrões	39,504518; -31,187712	Subtidal
35	FLO_SC_SCifs	Santa Cruz	Santa Cruz | Ilhéu Francisco	39,523814; -31,214148	Subtidal
36	FLO_SC_SCimvs	Santa Cruz	Santa Cruz | Ilhéu Maria Vaz	39,505833; -31,245	Subtidal
37	FLO_SC_SCipas	Santa Cruz	Santa Cruz | Ilhéu Pão de Açucar	39,500367; -31,170582	Subtidal
38	FLO_SC_SCpiem	Santa Cruz	Santa Cruz | Piscinas | Entre-marés	39,458842; -31,124608	Intertidal
39	FLO_SC_SCpcs	Santa Cruz	Santa Cruz | Ponta da Caveira	39,424187; -31,145587	Subtidal
40	FLO_SC_SCpis	Santa Cruz	Santa Cruz | Ponta do lhéu	39,509661; -31,19527	Subtidal
41	FLO_SC_SCpros	Santa Cruz	Santa Cruz | Ponta Ruiva | Oeste	39,495572; -31,152406	Subtidal
42	FLO_SC_SCpbbd	Santa Cruz	Santa Cruz | Porto da Baleia | Baía | Deep	39,463387; -31,127258	Subtidal
43	FLO_SC_SCpbbs1	Santa Cruz	Santa Cruz | Porto da Baleia | Baía | Shallow 1	39,463035; -31,128021	Subtidal
44	FLO_SC_SCpbbs2	Santa Cruz	Santa Cruz | Porto da Baleia | Baía | Shallow 2	39,463731; -31,12752	Subtidal
45	FLO_SC_SCpbbb	Santa Cruz	Santa Cruz | Porto da Baleia | Bóia flutuante	39,463035; -31,128021	Bóia
46	FLO_SC_SCpbem1	Santa Cruz	Santa Cruz | Porto da Baleia | Entre-marés 1	39,463518; -31,128256	Intertidal
47	FLO_SC_SCpbem2	Santa Cruz	Santa Cruz | Porto da Baleia | Entre-marés 2	39,463686; -31,128523	Intertidal
48	FLO_SC_SCpvs	Santa Cruz	Santa Cruz | Porto velho | Shallow	39,454305; -31,12449	Subtidal

**Table 3.** Information and location of the sampling sites on Corvo Island.

Location Nо	Location ID	Municipality	Locality / Latitude	Longitude	Littoral zone
1	COR_VC_VCaaem	Vila do Corvo	Vila do Corvo | Atrás do aeroporto	39,670289; -31,115366	Intertidal
2	COR_VC_VCms	Vila do Corvo	Vila do Corvo | Moldinho	39,668742; -31,120615	Subtidal
3	COR_VC_VCps	Vila do Corvo	Vila do Corvo | Pesqueiro	39,669127; -31,113446	Subtidal
4	COR_VC_VCps	Vila do Corvo	Vila do Corvo | Pesqueiro	39,669127; -31,113446	Subtidal
5	COR_VC_VCpas	Vila do Corvo	Vila do Corvo | Portinho da Areia	39,672838; -31,123437	Subtidal
6	COR_VC_VCpem	Vila do Corvo	Vila do Corvo | Porto da Casa | Entre-marés	39,671968; -31,110846	Intertidal
7	COR_VC_VCps	Vila do Corvo	Vila do Corvo | Porto da Casa | Subtidal	39,672729; -31,109214	Subtidal
8	COR_VC_VCpbem	Vila do Corvo	Vila do Corvo | Porto do Boqueirão | Entre-marés	39,669523; -31,112739	Intertidal
9	COR_VC_VCpbs	Vila do Corvo	Vila do Corvo | Porto do Boqueirão | Subtidal	39,668229; -31,112482	Subtidal

**Table 4.** Macroalgal species recorded from Flores Island, with information on relative abundance, origin and status.

**Phylum**	**Species (Accepted Name)**	**Number of records**	**Establishment Means**	**OccurrenceRemarks**
Chlorophyta	*Anadyomene stellata* (Wulfen) C.Agardh	13	Uncertain	
Chlorophyta	*Bryopsis cupressina* J.V.Lamouroux	2	Native	New record
Chlorophyta	*Bryopsis hypnoides* J.V.Lamouroux	4	Native	
Chlorophyta	*Bryopsis pennata* J.V.Lamouroux	1	Native	
Chlorophyta	*Bryopsis plumosa* (Hudson) C. Agardh	3	Native	
Chlorophyta	*Chaetomorpha aerea* (Dillwyn) Kützing	1	Native	
Chlorophyta	*Cladophora albida* (Nees) Kützing	3	Native	
Chlorophyta	*Cladophora coelothrix* Kützing	6	Native	
Chlorophyta	*Cladophora hutchinsiae* (Dillwyn) Kützing	2	Native	New record
Chlorophyta	*Cladophora lehmanniana* (Lindenberg) Kützing	5	Native	New record
Chlorophyta	*Cladophora prolifera* (Roth) Kützing	20	Native	
Chlorophyta	*Cladophoropsis membranacea* (Hofman Bang ex C.Agardh) Børgesen	1	Uncertain	
Chlorophyta	*Codium adhaerens* C.Agardh	18	Native	
Chlorophyta	*Codium decorticatum* (Woodward) M.A.Howe	3	Native	New record
Chlorophyta	Codium fragile subsp. fragile (Suringar) Hariot	5	Introduced	New record
Chlorophyta	*Derbesia marina* (Lyngbye) Solier	1	Native	
Chlorophyta	*Lychaete pellucida* (Hudson) M.J.Wynne	4	Native	New record
Chlorophyta	*Microdictyon umbilicatum* (Velley) Zanardini	31	Native	
Chlorophyta	*Ulva clathrata* (Roth) C.Agardh	3	Native	
Chlorophyta	*Ulva intestinalis* Linnaeus	8	Native	
Chlorophyta	*Ulva rigida* C.Agardh	10	Native	
Chlorophyta	*Valonia utricularis* (Roth) C.Agardh	3	Native	
Ochrophyta	*Ascophyllum nodosum* (Linnaeus) Le Jolis	7	Native	
Ochrophyta	*Carpomitra costata* (Stackhouse) Batters	2	Native	
Ochrophyta	*Cladostephus spongiosus* (Hudson) C.Agardh	23	Native	
Ochrophyta	*Colpomenia sinuosa* (Mertens ex Roth) Derbès & Solier	61	Native	
Ochrophyta	*Cutleria multifida* (Turner) Greville	4	Uncertain	
Ochrophyta	*Cutleria multifida* (Turner) Grevill, phase *Aglaozonia parvula* (Greville) Zanardini	2	Uncertain	New record
Ochrophyta	*Cystoseira foeniculacea* (Linnaeus) Greville	4	Native	
Ochrophyta	*Cystoseira humilis* Schousboe ex Kützing	1	Native	
Ochrophyta	*Dictyopteris polypodioides* (A.P.De Candolle) J.V.Lamouroux	2	Native	New record
Ochrophyta	*Dictyota bartayresiana* J.V.Lamouroux	4	Native	
Ochrophyta	*Dictyota cyanoloma* Tronholm, De Clerck, A.Gómez-Garreta & Rull Lluch	1	Native	New record
Ochrophyta	*Dictyota dichotoma* (Hudson) J.V.Lamouroux	3	Native	
Ochrophyta	*Halopteris filicina* (Grateloup) Kützing	54	Native	
Ochrophyta	*Halopteris scoparia* (Linnaeus) Sauvageau	61	Native	
Ochrophyta	*Hydroclathrus tilesii* (Endlicher) Santiañez & M.J.Wynne	1	Introduced	New record
Ochrophyta	*Leathesia marina* (Lyngbye) Decaisne	6	Uncertain	
Ochrophyta	*Lobophora variegata* (J.V.Lamouroux) Womersley ex E.C.Oliveira	11	Native	New record
Ochrophyta	*Myrionema strangulans* Greville	1	Native	
Ochrophyta	*Padina pavonica* (Linnaeus) Thivy	85	Native	
Ochrophyta	*Papenfussiella kuromo* (Yendo) Inagaki	1	Introduced	New record
Ochrophyta	*Petrospongium berkeleyi* (Greville) Nägeli ex Kützing	1	Native	New record
Ochrophyta	*Sargassum furcatum* Kützing	5	Native	New record
Ochrophyta	*Sargassum vulgare* C.Agardh, nom. illeg.	5	Native	
Ochrophyta	*Sphacelaria cirrosa* (Roth) C.Agardh	1	Native	New record
Ochrophyta	*Taonia atomaria* (Woodward) J.Agardh	6	Native	New record
Ochrophyta	*Zanardinia typus* (Nardo) P.C.Silva	15	Native	New record
Ochrophyta	*Zonaria tournefortii* (J.V.Lamouroux) Montagne	96	Native	
Rhodophya	*Acrosorium ciliolatum* (Harvey) Kylin	35	Native	
Rhodophya	*Amphiroa beauvoisii* J.V.Lamouroux	1	Native	
Rhodophya	*Amphiroa rigida* J.V.Lamouroux	5	Native	
Rhodophya	*Asparagopsis armata* Harvey	58	Introduced	
Rhodophya	*Asparagopsis armata* Harvey, phase *Falkenbergia rufolanosa* (Harvey) F.Schmitz	6	Introduced	
Rhodophya	*Asparagopsis taxiformis* (Delile) Trevisan	38	Native	
Rhodophya	*Bornetia secundiflora* (J.Agardh) Thuret	2	Native	
Rhodophya	*Botryocladia botryoides* (Wulfen) Feldmann	8	Native	New record
Rhodophya	*Callithamnion corymbosum* (J.E.Smith) Lyngbye	3	Native	
Rhodophya	*Callithamnion granulatum* (Ducluzeau) C.Agardh	2	Native	New record
Rhodophya	*Caulacanthus ustulatus* (Turner) Kützing	2	Uncertain	
Rhodophya	*Centroceras clavulatum* (C.Agardh) Montagne	14	Native	
Rhodophya	*Ceramium ciliatum* (J.Ellis) Ducluzeau	2	Native	
Rhodophya	*Ceramium cimbricum* H.E.Petersen	3	Native	
Rhodophya	*Ceramium derbesii* Solier ex Kützing	2	Native	
Rhodophya	*Ceramium echionotum* J.Agardh	1	Native	New record
Rhodophya	*Ceramium gaditanum* (Clemente) Cremades	2	Uncertain	
Rhodophya	*Ceramium virgatum* Roth	3	Native	
Rhodophya	*Ceratodictyon intricatum* (C.Agardh) R.E.Norris	2	Native	
Rhodophya	*Ceratodictyon scoparium* (Montagne & Millardet) R.E.Norris	1	Uncertain	New record
Rhodophya	*Chondracanthus acicularis* (Roth) Fredericq	11	Native	
Rhodophya	*Chondracanthus teedei* (Mertens ex Roth) Kützing	1	Native	New record
Rhodophya	*Chondria dasyphylla* (Woodward) C.Agardh	6	Uncertain	
Rhodophya	*Corallina ferreyrae* E.Y.Dawson, Acleto & Foldvik	7	Native	New record
Rhodophya	*Corallina officinalis* Linnaeus	18	Native	
Rhodophya	*Cruoria pellita* (Lyngbye) Fries	1	Native	
Rhodophya	*Cryptopleura ramosa* (Hudson) L.Newton	2	Native	New record
Rhodophya	*Ellisolandia elongata* (J.Ellis & Solander) K.R.Hind & G.W.Saunders	3	Native	
Rhodophya	*Erythrocystis montagnei* (Derbès & Solier) P.C.Silva	2	Native	New record
Rhodophya	*Gelidium corneum* (Hudson) J.V.Lamouroux	10	Native	
Rhodophya	*Gelidium microdon* Kützing	4	Native	
Rhodophya	*Gelidium pusillum* (Stackhouse) Le Jolis	12	Native	
Rhodophya	*Gelidium spinosum* (S.G.Gmelin) P.C.Silva	11	Native	
Rhodophya	*Gigartina pistillata* (S.G.Gmelin) Stackhouse	1	Native	
Rhodophya	*Gracilariopsis longissima* (S.G.Gmelin) Steentoft, L.M.Irvine & Farnham	2	Native	
Rhodophya	*Grateloupia filicina* (J.V.Lamouroux) C.Agardh	10	Native	
Rhodophya	*Griffithsia corallinoides* (Linnaeus) Trevisan	1	Uncertain	
Rhodophya	*Griffithsia devoniensis* Harvey	1	Native	New record
Rhodophya	*Gymnogongrus crenulatus* (Turner) J.Agardh	14	Native	
Rhodophya	*Gymnogongrus griffithsiae* (Turner) C.Martius	3	Native	
Rhodophya	*Gymnothamnion elegans* (Schousboe ex C.Agardh) J.Agardh	2	Native	
Rhodophya	*Halurus flosculosus* (J.Ellis) Maggs & Hommersand	1	Native	
Rhodophya	*Hypnea musciformis* (Wulfen) J.V.Lamouroux	19	Uncertain	
Rhodophya	*Hypoglossum hypoglossoides* (Stackhouse) Collins & Hervey	9	Native	New record
Rhodophya	*Jania capillacea* Harvey	1	Native	New record
Rhodophya	*Jania crassa* J.V.Lamouroux	2	Native	New record
Rhodophya	*Jania longifurca* Zanardini	8	Uncertain	
Rhodophya	*Jania rubens* (Linnaeus) J.V.Lamouroux	6	Native	
Rhodophya	*Jania virgata* (Zanardini) Montagne	13	Uncertain	
Rhodophya	*Kallymenia reniformis* (Turner) J.G.Agardh	1	Native	
Rhodophya	*Laurencia obtusa* (Huds.) J.V.Lamouroux	8	Native	
Rhodophya	*Laurencia viridis* Gil-Rodríguez & Haroun	3	Macaronesian endemism	New record
Rhodophya	*Laurenciella marilzae* (Gil-Rodríguez, Sentíes, Díaz-Larrea, Cassano & M.T.Fujii) Gil-Rodríguez, Sentíes, Díaz-Larrea, Cassano & M.T.Fujii	4	Native	New record
Rhodophya	*Leptosiphonia fibrillosa* (Agardh) A.M.Savoie & G.W.Saunders	1	Native	
Rhodophya	*Lomentaria articulata* (Hudson) Lyngbye	12	Native	
Rhodophya	*Mesophyllum expansum* (Philippi) Cabioch & M.L.Mendoza	1	Native	New record
Rhodophya	*Millerella tinerfensis* (Seoane-Camba) S.M.Boo & J.M.Rico	3	Macaronesian endemism	
Rhodophya	*Nemalion elminthoides* (Velley) Batters	5	Native	
Rhodophya	*Neoizziella divaricata* (C.K.Tseng) S.-M.Lin, S.-Y.Yang & Huisman	5	Introduced	New record
Rhodophya	*Osmundea hybrida* (A.P.de Candolle) K.W.Nam	4	Native	
Rhodophya	*Osmundea pinnatifida* (Hudson) Stackhouse	10	Native	
Rhodophya	*Peyssonnelia squamaria* (S.G.Gmelin) Decaisne ex J.Agardh	6	Native	
Rhodophya	*Phyllophora crispa* (Hudson) P.S.Dixon	3	Native	
Rhodophya	*Platoma cyclocolpum* (Montagne) F.Schmitz	42	Native	New record
Rhodophya	*Plocamium cartilagineum* (Linnaeus) P.S.Dixon	23	Native	
Rhodophya	*Polysiphonia opaca* (C.Agardh) Moris & De Notaris	2	Native	
Rhodophya	*Polysiphonia stricta* (Mertens ex Dillwyn) Greville	1	Native	
Rhodophya	*Pterocladiella capillacea* (S.G.Gmelin) Santelices & Hommersand	42	Native	
Rhodophya	*Rhodymenia holmesii* Ardissone	14	Native	
Rhodophya	*Schimmelmannia schousboei* (J.Agardh) J.Agardh	1	Native	
Rhodophya	*Schizymenia apoda* (J.Agardh) J.Agardh	1	Native	New record
Rhodophya	*Schottera nicaeensis* (J.V.Lamouroux ex Duby) Guiry & Hollenberg	2	Uncertain	
Rhodophya	*Sphaerococcus coronopifolius* Stackhouse	20	Native	
Rhodophya	*Spyridia filamentosa* (Wulfen) Harvey	3	Native	
Rhodophya	*Stenogramma interruptum* (C.Agardh) Montagne	1	Native	
Rhodophya	*Symphyocladia marchantioides* (Harvey) Falkenberg	1	Introduced	
Rhodophya	*Taenioma nanum* (Kützing) Papenfuss	1	Native	New record
Rhodophya	*Tenarea tortuosa* (Esper) Me.Lemoine	1	Native	
Rhodophya	*Vertebrata fruticulosa* (Wulfen) Kuntze	2	Native	
Rhodophya	*Vertebrata fucoides* (Hudson) Kuntze	2	Uncertain	
Rhodophya	*Vertebrata thuyoides* (Harvey) Kuntze	1	Native	New record

**Table 5.** Summary of the macroalgal flora of Flores Island, with information on the species origins and status.

Phyllum	Order	Family	Specimens Number	Total taxa	Total species	Native	Introduced	Uncertain	Macaronesian endemism	New record
Rhodophyta	14	33	789	120	80	65	3	10	2	20
Chlorophyta	3	9	216	35	22	19	1	2		6
Ochrophyta	7	12	682	41	26	22	2	2		11
Total	24	54	1687	196	128	106	6	14	2	37

**Table 6.** Macroagal species recorded from Corvo Island, with information on relative abundance, origin and status.

**Phylum**	**Species (Accepted Name)**	**Number of records**	**Establishment Means**	**OccurrenceRemarks**
Chlorophyta	*Chaetomorpha linum* (O.F.Müller) Kützing	1	Native	
Chlorophyta	*Cladophora coelothrix* Kützing	1	Native	
Chlorophyta	*Cladophora hutchinsiae* (Dillwyn) Kützing	1	Native	New record
Chlorophyta	*Cladophora laetevirens* (Dillwyn) Kützing	1	Uncertain	
Chlorophyta	*Cladophora prolifera* (Roth) Kützing	2	Native	
Chlorophyta	Codium fragile subsp. fragile (Suringar) Hariot	2	Introduced	
Chlorophyta	*Microdictyon umbilicatum* (Velley) Zanardini	29	Native	New record
Chlorophyta	*Valonia utricularis* (Roth) C.Agardh	1	Native	New record
Ochrophyta	*Carpomitra costata* (Stackhouse) Batters	1	Native	New record
Ochrophyta	*Cladostephus spongiosus* (Hudson) C.Agardh	1	Native	New record
Ochrophyta	*Colpomenia sinuosa* (Mertens ex Roth) Derbès & Solier	18	Native	
Ochrophyta	*Cutleria multifida* (Turner) Greville	3	Uncertain	New record
Ochrophyta	*Dictyopteris polypodioides* (A.P.De Candolle) J.V.Lamouroux	3	Native	New record
Ochrophyta	*Halopteris filicina* (Grateloup) Kützing	31	Native	New record
Ochrophyta	*Halopteris scoparia* (Linnaeus) Sauvageau	15	Native	
Ochrophyta	*Leathesia marina* (Lyngbye) Decaisne	1	Uncertain	New record
Ochrophyta	*Lobophora variegata* (J.V.Lamouroux) Womersley ex E.C.Oliveira	8	Native	New record
Ochrophyta	*Padina pavonica* (Linnaeus) Thivy	32	Native	
Ochrophyta	*Sargassum furcatum* Kützing	2	Native	New record
Ochrophyta	*Taonia atomaria* (Woodward) J.Agardh	5	Native	New record
Ochrophyta	*Zonaria tournefortii* (J.V.Lamouroux) Montagne	33	Native	
Rhodophyta	*Acrosorium ciliolatum* (Harvey) Kylin	28	Native	New record
Rhodophyta	*Asparagopsis armata* Harvey	23	Introduced	
Rhodophya	*Asparagopsis armata* Harvey, phase *Falkenbergia rufolanosa* (Harvey) F.Schmitz	1	Introduced	
Rhodophyta	*Asparagopsis taxiformis* (Delile) Trevisan	13	Native	New record
Rhodophyta	*Carradoriella denudata* (Dillwyn) A.M.Savoie & G.W.Saunders	1	Uncertain	
Rhodophyta	*Caulacanthus ustulatus* (Mertens ex Turner) Kützing	1	Uncertain	New record
Rhodophyta	*Chondracanthus acicularis* (Roth) Fredericq	2	Native	
Rhodophyta	*Chondria capillaris* (Hudson) M.J.Wynne	1	Native	
Rhodophyta	*Corallina officinalis* Linnaeus	3	Native	
Rhodophyta	*Erythrodermis traillii* (Holmes ex Batters) Guiry & Garbary	1	Uncertain	
Rhodophyta	*Gelidium pusillum* (Stackhouse) Le Jolis	2	Native	
Rhodophyta	*Gigartina pistillata* (S.G.Gmelin) Stackhouse	1	Native	
Rhodophyta	*Gymnogongrus crenulatus* (Turner) J.Agardh	1	Native	New record
Rhodophyta	*Gymnogongrus griffithsiae* (Turner) C.Martius	5	Native	New record
Rhodophyta	*Jania virgata* (Zanardini) Montagne	8	Uncertain	New record
Rhodophyta	*Nemalion elminthoides* (Velley) Batters	1	Native	
Rhodophyta	*Osmundea pinnatifida* (Hudson) Stackhouse	2	Native	
Rhodophyta	*Plocamium cartilagineum* (Linnaeus) P.S.Dixon	4	Native	New record
Rhodophyta	*Pterocladiella capillacea* (S.G.Gmelin) Santelices & Hommersand	6	Native	
Rhodophyta	*Schottera nicaeensis* (J.V.Lamouroux ex Duby) Guiry & Hollenberg	1	Uncertain	
Rhodophyta	*Sphaerococcus coronopifolius* Stackhouse	3	Native	New record
Rhodophyta	*Spyridia filamentosa* (Wulfen) Harvey	2	Native	New record
Rhodophyta	*Vertebrata fruticulosa* (Wulfen) Kuntze	1	Native	

**Table 7.** Summary of the macroalgal flora of Corvo Island, with information on the species origins and status.

Phyllum	Order	Family	Specimens Number	Total taxa	Total species	Native	Introduced	Uncertain	New record
Rhodophyta	7	16	136	30	22	16	1	5	9
Chlorophyta	3	4	42	9	8	7	1	0	4
Ochrophyta	6	9	212	17	13	11	0	2	9
Total	16	29	390	56	43	34	2	7	22
